# Origin and age of the causative mutations in *KLC2*, *IMPA1*, *MED25* and *WNT7A* unravelled through Brazilian admixed populations

**DOI:** 10.1038/s41598-018-35022-1

**Published:** 2018-11-08

**Authors:** Allysson Allan de Farias, Kelly Nunes, Renan Barbosa Lemes, Ronald Moura, Gustavo Ribeiro Fernandes, Uirá Souto Melo, Mayana Zatz, Fernando Kok, Silvana Santos

**Affiliations:** 1 Department of Genetics and Evolutionary Biology, Biosciences Institute, University of São Paulo (USP), São Paulo, SP Brazil; 2 Department of Genetics, Federal University of Pernambuco (UFPE), Recife, PE Brazil; 3 Department of Chemistry, Institute of Chemistry, University of São Paulo (USP), São Paulo, Brazil; 4 Human Genome and Stem-Cell Center, Institute of Biosciences, University of São Paulo (USP), São Paulo, Brazil; 5 Department of Neurology, Faculty of Medicine (FMUSP), University of São Paulo (USP), São Paulo, Brazil; 6 Department of Biology, State University of Paraíba (UEPB), Campina Grande, PB Brazil

## Abstract

The mutation age and local ancestry of chromosomal segments harbouring mutations associated with autosomal recessive (AR) disorders in Brazilian admixed populations remain unknown; additionally, inbreeding levels for these affected individuals continue to be estimated based on genealogical information. Here, we calculated inbreeding levels using a runs of homozygosity approach, mutation age and local ancestry to infer the origin of each chromosomal segments containing disorder-causing mutations in *KLC2*, *IMPA1*, *MED25* and *WNT7A*. Genotyped data were generated from 18 patients affected by AR diseases and combined to the 1000 genome project (1KGP) and Simons genome diversity project (SGDP) databases to infer local ancestry. We found a major European contribution for mutated haplotypes with recent mutation age and inbreeding values found only in Native American and Middle East individuals. These results contribute to identifying the origin of and to understanding how these diseases are maintained and spread in Brazilian and world populations.

## Introduction

Local ancestry inference (LAI) is broadly used to identify the origin of many genetic diseases in worldwide populations by means of haplotype-based and single-marker genotype approaches^1–4^. LAI can be used to estimate the continental and subcontinental putative origin for an individual at a given genomic position, and it is applied in many studies related to the demographic history of populations^5,6^, in association studies^7,8^ and in pharmacogenomics^9,10^, clarifying the information about parental genetic contributions to the complex mosaic chromosomal segments of admixed populations^11^. In admixed human populations, for example, LAI has been used to increase the potential of admixture-mapping analysis for Mendelian and complex diseases, including ancestry-specific allele frequency estimation and genotype-phenotype association studies^7^.

The genome of individuals presents locus-specific ancestry blocks resulting from parental segments broken by recombination events at approximately twenty generations^11^. The Brazilian demographic history is made up by the genetic contributions of Native Americans, Europeans migrants since the early sixteenth century, and the influx of enslaved Africans that intensified in the seventeenth century, originating the current tri-hybrid population^11,12^. The Northeast (NE) Brazilian population represents 28% of the total population of the country, having the lowest Human Development Index (HDI)^13^. This region includes many small communities, with less than 25,000 inhabitants, located in the backlands that were conquered by Europeans during the 17^th^ century.

These small communities were historically considered to be geographic isolates or semi-isolates, usually showing high consanguinity levels, with Wright’s fixation index F-values of approximately 1%^14,15^, which are approximately 10-fold higher than those observed for the Southern Brazilian region and are similar to those observed for Middle Eastern countries^16,17^. The population average inbreeding levels were only estimated based on genealogical information and taking into account the number of marriages between closely related individuals for these Northeast Brazilian communities. Thus, no molecular genetic studies have been performed.

High inbreeding levels in a population increase the frequencies of individuals carrying rare variants in homozygous state, known as genetic drift. Four autosomal recessive (AR) genetic disorders were firstly described by our group in Northeast Brazilian communities: (1) AR intellectual disability related to a homozygous missense mutation in *MED25* (MIM 610197, chr19:50,332,240 GRCh37/hg19)^18^, (2) AR intellectual disability related to a homozygous duplication of 5-bp in *IMPA1* (MIM 602064, chr8: 82,583,247 GRCh37/hg19)^19^, and (3) a AR fibular and ulnar absence related to a homozygous variant in *WNT7A* (MIM 613005, chr3:13,860,557 GRCh37/hg19)^20^. Up to now, *IMPA1*, *MED25* and *WNT7A* pathogenic variants detected in the region have been reported only in these Northeast Brazilian communities.

The fourth disease, spastic paraplegia, optic atrophy, and neuropathy (SPOAN) syndrome (MIM #609541)^21^, is a severe neurodegenerative disease caused by a 216-bp deletion in the regulatory region of *KLC2* (chr11:66,024,557_66,024,773del GRCh37/hg19), affecting more than 70 reported individuals in the Northeast, Southeast and Southern populations of Brazil^22^. Interestingly, this *KLC2* mutation was also detected in two affected siblings from Egypt. Thus, the *KLC2* mutation is not restricted to Brazil; it is supposed that the mutation origin was allochthonous, but it is not clear where and when it arose.

Here, we estimate the age and based on local ancestry inference (LAI) methods, infer the origin of the chromosomal segments containing causative mutations in *KLC2*, *IMPA1*, *MED25* and *WNT7A* genes. To determine the role of inbreeding in the incidence of these AR diseases, we measure inbreeding using the runs of homozygosity approach (ROH), which quantifies homozygous segment size in individuals, inherited by descent (IBD). Through the analysis of admixed individuals affected by AR diseases, our aim is to contribute to identifying their origin and to understanding how these diseases are maintained and spread in Brazilian and world populations.

## Results

### Mutation age

**Figure 1:**
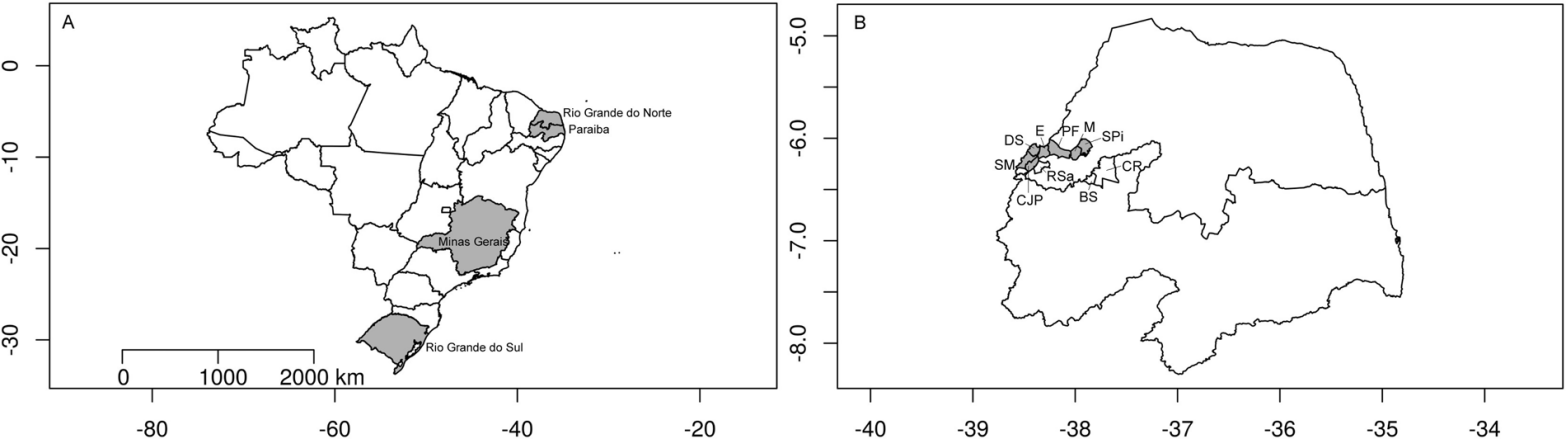
Location of affected Brazilian individuals. (**A**) States in grey have affected individuals. (**B**) State of Paraiba below, inside Catole do Rocha - CR and Brejo dos Santos - BS municipality; State of Rio Grande do Norte with SPOAN-affected individuals, RN Brazilian cluster (grey). Serrinha dos Pintos - SR; Martins - M; Pau dos Ferros - PF, Encanto - E, Doutor Severiano - DR, Sao Miguel - SM, Coronel Joao Pessoa - CJP. Affected with Santos syndrome in Riacho de Santana - Rsa (blank).

The most recent common ancestor (MRCA) is estimated assuming the shared length of common pair of haplotypes harbouring the homozygous mutation region. The ancestor of known affected individuals with the mutation in *MED25* represents the entrance of the mutation in the *Catole do Rocha* municipality (Fig. 1), which was estimated assuming a correlated genealogy of 8.7 generations (95% confidence interval (CI): 3.4–22.9). The *IMPA1* mutation arose in the backlands approximately eight generations ago, according to the genealogical records^19^. However, the mutation age estimate was not accurate due to the small number of affected individuals (n = 2) and the high CI (2.2–25). The *WNT7A* mutation accounts for the entrance of the derived allele in 2.9 generations (95% CI: 1.3–7) in the *Riacho de Santana* municipality. SPOAN syndrome showed quite different results due to the presence of affected individuals outside the Rio Grande do Norte cluster with a shared common haplotype. The homozygous mutation in *KLC2* spread in Rio Grande do Norte cluster for approximately 16 generations (95% CI: 9–28.6), and considering the haplotype of affected patients from two different states in Brazil and one Egyptian family, it was estimated as 19.4 generations (95% CI: 12.2–31.1). Table 1 summarizes all these results.

**Table 1:** Mutation age for each autosomal recessive disease.

Mutated gene	Average length of chromosome (cM)	Markers on chromosome	Age in generations (correlated genealogy)	95% confidence interval
*KLC2*	149.46	32,135	19.4	12.1–30.9
*MED25*	108.82	14,364	8.7	3.4–22.9
*WNT7A*	218.14	45,171	2.9	1.2–7.3

### Global ancestry

**Figure 2:**
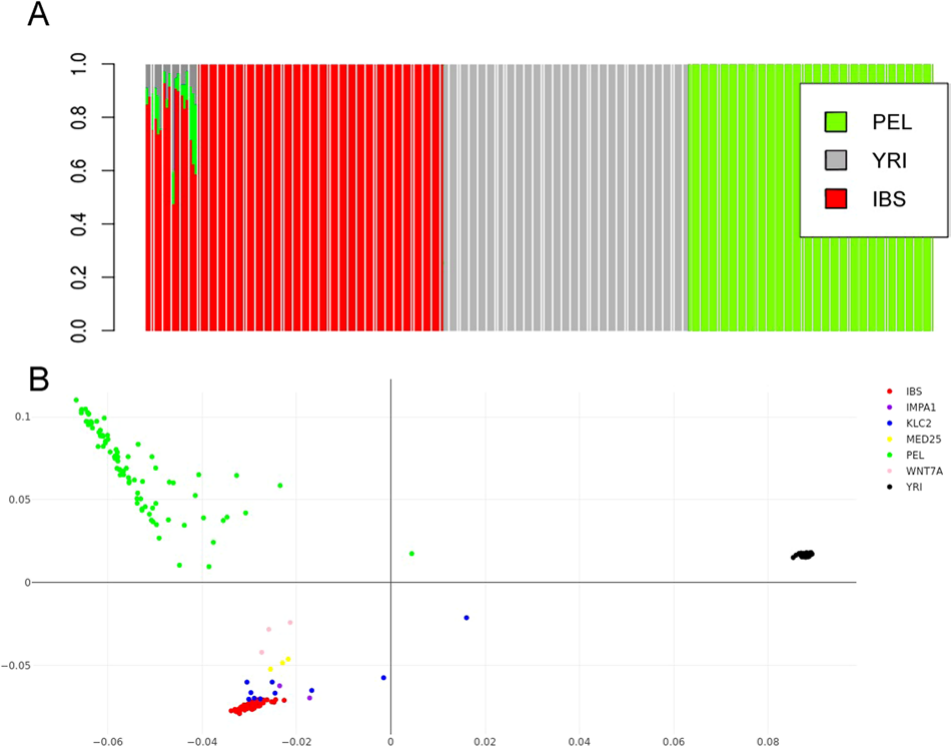
Principal component analysis and ADMIXTURE results obtained using the IBS, YRI and PEL sets of populations.

Principal component analysis (PCA), considering Peruvians from Lima (PEL) as proxy Native Americans, revealed that all affected individuals clustered close to the European populations. Affected individuals with *MED25* and *IMPA1* mutations were tightly clustered near Europeans, who were similar to the affected individuals with the *KLC2* mutation, whereas three affected individuals with *WNT7A* mutation were located slightly outside of the Spanish (IBS) cluster (Fig. 2A).

The European signal obtained using the same admixed Native American population was also supported by supervised ADMIXTURE outcomes, revealing a more than two-thirds European component for almost all affected individuals; the mean ancestry ± standard deviation was 0.8497 ± 0.1069 assuming K = 3 after the cross-validation procedure (Fig. 2B). Only one *KLC2*-affected individual from MG (Southeast Brazilian region) showed a half contribution of an African component for their ancestry (0.4056). The affected Egyptian individual had a one-fourth Yoruba (YRI) contribution (0.2410). Individuals affected for *MED25*, *IMPA1* and *WNT7A* presented a mean European ancestry of 0.7839 ± 0.0241, 0.8662 ± 0.0087, and 0.7174 ± 0.6120, respectively. The outcomes of PCA and ADMIXTURE obtained using three other datasets are present in Supplementaries Figs 1 and 2.

### Ancestry for *KLC2*, *IMPA1*, *MED25*, and *WNT7A* haplotypes harbouring mutations

**Figure 3:**
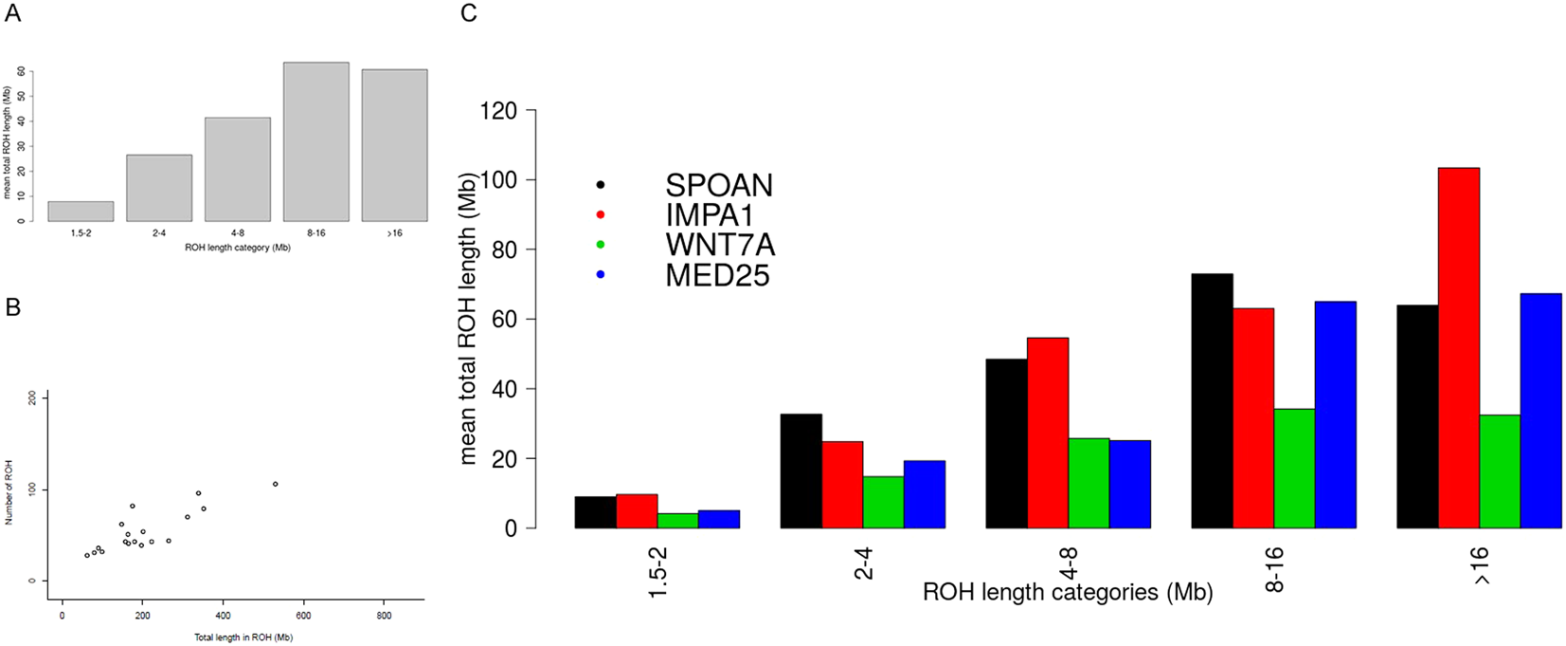
Molecular inbreeding estimates of ROH. (**A**) The mean of total length categories compared to the mean total ROH length for all affected individuals. (**B**) The total length in ROH compared to the number of ROHs for all affected individuals. (**C**) The mean of total length categories compared to the mean total ROH length, separated by disease group.

LAI using 1KGP and SGDP references merged with AR dataset revealed a considerable European proportion, agreeing with the outcomes from ADMIXTURE analysis, which reached over two-thirds for the European component for affected individuals that were homozygous for *KLC2*, *IMPA1*, *MED25* and *WNT7A* mutations (Supplementaries Figs 3–6).

The use of PEL as proxy Native Americans showed that haplotypes containing the *KLC2* mutation presented European ancestry. A mean segment length of 104.63 Mb (104.12 cM) in genomes of the affected Rio Grande do Norte state Brazilian cluster was higher than the 93.54 Mb (92.90 cM) length among individuals from all clusters: Rio Grande do Norte (RN), Minas Gerais (MG), Rio Grande do Sul (RS) states and Egypt (EG). The mean European segment length for *MED25* was 11.35 Mb (31.60 cM), and for *IMPA1*, it was 113.79 Mb (116.21 cM). The *WNT7A*-affected individuals revealed a Native American locus-specific ancestry; the mean segment length was 24.54 Mb (39.83 cM). The results of three other sets using Japanese (JPT) and Han Chinese (CHB) populations from 1KGP and Native American references from SGDP are present in Supplementary Table 1.

### Molecular inbreeding estimates

Inbreeding coefficient estimate from ROH (Froh) results revealed values near 1% for *IMPA1-*, *KLC2-* and *MED25*-affected individuals; for *WNT7A*, it was below the 0.5% threshold (Table 2). We divided ROH results into three subclasses according to their lengths, long (>8 Mb), intermediate (>2–8 Mb) and short (1.5–2 Mb) tracts, which confirmed a high mean of the total ROH length for the long tracts category that reached more than 50 Mb, revealing high levels of recent inbreeding, as expected for AR-affected individuals (Fig. 3A). The total number of long genomic stretches exceeding 1.5 Mb presented in all affected individuals showed values ranging between 25 and 90 segments. The observed cumulative ROH lengths were particularly high, varying from 50 to 300 Mb for each affected disease group (Fig. 3B). Individuals carrying *KLC2* and *IMPA1* mutations presented ROH over 350 Mb long, whereas the genome of individuals carrying *WNT7A* and *MED25* mutations had smaller ROH (below 100 Mb).

**Table 2:** Mean, median and variation of Froh estimated for each disease, considering ROH above 1.5 Mb.

	Mean	Median	Var(Froh)
*KLC2*	0.0840	0.067	0.002
*IMPA1*	0.0806	0.080	0.004
*MED25*	0.0623	0.059	0.001
*WNT7A*	0.0435	0.044	0.025

Individuals affected with mutations in *KLC2*, *IMPA1* and *MED25* showed a similar pattern for average total ROH length when the long subset was considered, with values larger than 60 Mb, although one affected individual with *WNT7A* presented an estimated average total ROH length half of this value; the intermediate and short categories exhibited a tendency for *WNT7A* and *MED25* to have almost identical average lengths, as did those as for affected *KLC2* and *IMPA1* (Fig. 3C).

## Discussion

The genetic outcomes of global ancestry inference for our samples corroborate the historical registers of the Brazilian Colonial Period (1500–1815), showing that the European invasion, the Native American decline and the admixture events between them in the backlands might have led to a high European contribution to the genetic constitution of the affected individuals in NE Brazil studied here, reaching more than 90% in some cases. The European contribution is higher than the African and Native American contributions as expected^23–25^, and is above the threshold observed for NE Brazilian admixed populations^26–28^. Individuals living in the backlands probably have major genomic contributions from Iberian individuals, according to historical records, and can carry deleterious genetic variations found in European individuals^29^. Beyond Iberians, other Europeans, such as French and Dutch populations, groups of Sephardic Jews and Moors persecuted by the Inquisition, ancient Native Americans and recent Africans^30^, gave rise to NE Brazilian backland populations.

The mutation age for *KLC2* homozygous deletion is approximately 485 years and is older than those for two of the other mutations, *MED25* (~218 years) and *WNT7A* (~73 years). LAI revealed a European contribution to the *KLC2* locus in individuals carrying the pathogenic mutation. According to the historical background, the families that carry the mutations probably originated from either European or Southern American current territories. The *KLC2* mutation carriers probably inhabited NE Brazilian backlands during the period of Dutch invasion along the coast (1630–1654). The origin of this mutation might be in the Iberian peninsula during the persecution of Sephardic Jews and Moors by the Inquisition (1542). The evidence of two affected brothers in Egypt sharing the same mutation indicates that the mutation originated inside Europe or the Middle East. However, no direct historical link between Egypt and Brazil was found, despite the fact that many North African communities registered events of migration between Europe and Middle East along the coast of the Mediterranean Sea. Our hypothesis points to the origin of mutation lying in the Andalusia region in the South of the Iberian Peninsula, arising from ethnic groups persecuted as Moorish and Sephardic Jews, which arrived in Brazil during the 17^th^ century (Supplemental Data 1). Furthermore, individuals living in the Iberian Peninsula and Middle East might be at risk for the *KLC2* 216-bp deletion.

Our findings are similar to those of the Machado-Joseph disease, suggesting an origin of the mutation in the Portugal mainland, which was introduced to the Azores Islands by Portuguese settlers, spreading the mutation to colonies such as Brazil and India^31,32^. A cluster of 19 individuals with this disease was identified in Lagoa community, which is near to the *KLC2* cluster and three other families here investigated^33^. Historically, other neurodegenerative disorders were found in Brazilian families, including SPG8 and X-linked SPG, among others highly spread in Brazil^34^; there are also documented affected individuals living in the Iberian region^35^, a population that represents our ancient colonizers.

The population of *KLC2* mutation carriers suffered a severe bottleneck in the NE region cluster, with a demographic explosion from approximately 20,000 to 250,000 inhabitants between the end of the 19^th^ to 20^th^ century according to the 1872 and 2000 census published by the Brazilian Institute of Geography and Statistics. This demographic explosion was marked by cultural practices of consanguineous marriage, which raised the number of affected individuals due to genetic drift. The founder effect associated with endogamy explains the mutated allele’s dispersion being conserved at a high frequency. Estimates of genomic homozygosity were consistent with previously inferred inbreeding coefficient for pedigrees from NE backlands. The Froh showed that average values of *KLC2* (0.0840) and *IMPA1* (0.0806) groups were higher than those of Native Americans (Table 2), suggesting that these affected individuals are extremely inbred. The cumulative length and number of ROHs in affected individuals observed using single nucleotide polymorphism (SNP) array data showed a picture found in Central/South Asian populations, with communities clearly known to be highly consanguineous such as the Balochi^36^, and in Brazilian *quilombo* communities^37^. The distribution of different length categories of ROH were completely proportional to other results already published, suggesting that affected individuals with rare recessive disorders had recent events of endogamy compared to worldwide individuals in small isolated populations^38,39^.

The other three mutations arose more recently in Brazilian backlands. The *MED25* mutation emerged over ten generations ago, *IMPA1* eight generations ago and *WNT7A* four generations ago. *MED25* and *IMPA1* haplotypes showed a European ancestry; however, the mutation might have a Brazilian origin since, to date, no affected individual with the same mutation has been found elsewhere. Other small communities with high consanguinity levels from the Middle East and Central and South Asia carry loss-of-function and missense mutations that might cause intellectual disabilities, but nothing is known about their mutation age^40^.

Interestingly, LAI for *WNT7A* showed a Native American ancestry-specific locus, suggesting that the mutation originated in the Brazilian backlands. The mutation age of only four generations was probably due the small number of known affected individuals sharing the same mutation in this Native American haplotype. This autochthonous mutation probably randomly arose in a Native American haplotype after contact with Europeans and Africans. Other mutations with Native American ancestry have been described, such as the deleterious variant in *ACTN1*, which expresses congenital macrothrombocytopenia^41^, and the spinocerebellar ataxia type 10 in *ATXN10* that causes progressive cerebellar ataxia and epilepsy^42^.

In short, our evidence pinpoints the need to understand the history of genetic diseases based on LAI to identify at-risk populations, contributing to corroborating the historical record of communities with high consanguinity levels and their recessive autosomal diseases. We identified three autochthonous mutations and one allochthonous mutation, which might be spread from the populations living at the borders of Mediterranean Sea. In addition, for admixture populations, the ‘LAI and mutation age’ approach can elucidate the demographic trajectory of genetic mutations in populations.

## Methods

### Autosomal Recessive Dataset (AR)

We genotyped 18 affected individuals sampled from distinct family branches (Table 3). Ten affected individuals with cryptic relatedness were affected by *KLC2* mutation: seven from different communities in Rio Grande do Norte (RN) state, one from Rio Grande do Sul (RS) state, one from Minas Gerais (MG) state and one from Tanta - Gharbia Governorate, in Egypt (EG); three first cousins with *MED25*-associated intellectual disability were from Paraiba (PB) state; two second cousins with intellectual disability related to *IMPA1* were also from PB; and three first cousins with *WNT7A*-related skeletal dysplasia (Santos syndrome) were from RN (Fig. 1). Further genealogical details are provided elsewhere^18,19,22,43,44^. All affected individuals and families were orally informed about the strict scientific use and guaranteed anonymity of sampled data.

**Table 3:** AR-affected individuals with genetic disorders.

Disease	Gene	Number of genotyped individuals	Location	Estimated number of affected individuals
SPOAN syndrome	*KLC2*	7	RN	75^22^
		1	MG	
		1	RS	
		1	EG	
Intellectual disability	*MED25*	3	PB	7^18^
Intellectual disability	*IMPA1*	2	PB	9^19^
Santos syndrome	*WNT7A*	3	RN	5^43^

### Public Datasets

To infer global and local ancestry, we selected unrelated individuals from parental world populations available in the 1000 Genomes Project (1KGP)^45^: (1) Peruvians from Lima (PEL), representing admixed Native Americans (77.3% Native American component^46^); (2) Spanish individuals (IBS) representative of the European component; and (3) Yoruba individuals (YRI) for the African component. Due to the non-availability of Native American samples compatible with our set of SNPs, we tested the robustness of the results in three different populational sets: (a) IBS and YRI with Japanese (JPT) and (b) IBS and YRI with Han Chinese (CHB), both being genetically close to Native Americans in the 1KGP database; (c) European, African, and Native American populations present in the Simons Genome Diversity Project (SGDP)^47^ (see details in Supplemental Data 2).

### Data cleaning and filter

#### AR Dataset

Samples were genotyped in the present study using the Axiom World Array LAT (817,810 SNP markers) according to the manufacturer’s instructions (Affymetrix/Thermo-Fisher Scientific)^48^. Quality filters were applied using Affymetrix Genotyping Console (version 4.0) and Affymetrix SNPolisher package in R (version 1.5.2) to remove batch and Mendelian errors, high missing call rates for individuals (>5%) and sex differences in allele frequency, Removal of the centromere and 2 Mb of each chromosome telomere was performed across all sampled individuals. After the initial filtering process, the number of SNPs was reduced to 656,743.

#### Public Datasets

Biallelic markers from 1KGP and SGDP datasets were merged with genotyped data using PLINK v1.9^49^. The merged data with the 1KGP dataset considered a deviation from Hardy-Weinberg equilibrium (HWE) less than 10^−8^, a minor allele frequency (MAF) greater than 1%, and a missing call rate below 1%. The final merged dataset included 493,780 SNPs across 273 individuals from IBS, YRI, PEL, and our data. The same threshold was applied for the other two sets of populations: (a) 318 individuals and 488,548 SNPS for JPT and (b) 318 individuals and 488,706 SNPs for CHB. The selected threshold for the SGDP set (c) was a missing call rate below 25% for each SNP due to low overlap of SNPs from our data and the parental population.

#### Most recent common ancestor (MRCA)

The MRCA is the last common ancestor for a given set of individuals who carry an ancestral haplotype, and the age of the MRCA is equivalent to the estimated mutation age (in number of generations) in a population. The mutation age was estimated using the Gamma method^50^ based on the length of shared ancestral haplotypes between AR-affected individuals, which was developed especially for small samples sizes and high density SNPs. The samples were chosen based on correlated genealogy, with each one from different nuclear families. The haplotypes of affected individuals grouped by disease were delimited by continuous shared markers. The option for correlated genealogy results was used due to the possibility of the mutation age being more recent than MRCA, and the adopted confidence interval parameter (CI) was 95%.

#### ROH

Data were filtered for inbreeding coefficient estimate from ROH (Froh)^38^ using the selected variants with a) a missing call rate below 1% for each SNP; b) deviation from Hardy-Weinberg equilibrium (HWE) below 10–8; and c) a minor allele frequency (MAF) larger than 1% using PLINK v1.9^49^. The sliding windows presented a minimum of 25 SNPs, with a step-by-step size of 10 SNPs using PLINK. We considered a minimum length of 1.5 Mb for ROH.

Long runs of homozygosity (ROH) were identified using PLINK. The method used by McQuillan *et al*.^38^ was used to calculate the inbreeding coefficient, assuming a minimum length of 1500 kb for ROH stretches. The retained total lengths of ROH for affected individuals were summed up for division by the total length of the autosomal genome covered by the SNP array, resulting in the Froh estimate. We followed parameters tested elsewhere^37^.

#### Global and local ancestry analysis

The population relationship was investigated by principal component analysis (PCA) using PLINK 1.9^49^. ADMIXTURE v1.23^51^ analysis was performed to infer the genetic global ancestry for each affected individual used a Bayesian model-based algorithm with a tri-hybrid population reference. The genotype markers were pruned using a threshold of R2 ≥ 0.1 and a 50-SNP window advancing by ten SNPs in PLINK. We perform a supervised analysis with K = 3 and 2000 bootstrap replicates.

The inference of haplotypes for all biallelic genetic variants of the AR Dataset and the Public Dataset was performed using SHAPEIT2 software^52^, and 1092 samples from 1KGP Phase 3 were used as a reference panel^45^. After obtained the phased genotypes, we proceed with LAI for all SNPs using the RFMix algorithm^3^. RFMix is a discriminative approach that uses conditional random fields based on reference panel data on an SNP-by-SNP basis; additionally, the algorithm incorporates an expectation-maximization (EM) to refine the LAI. We used the PopPhased option using default parametrization and reference panels in EM. The locus-specific ancestry obtained by RFMix show us the putative origin for the common haplotype shared when associated with an established mutation age.

Given that the haplotypes lengths are known, both haplotype lengths of each individual were summed up and divided by two. A mean segment length for the locus-specific ancestry harbouring the mutation was calculated assuming the segment length obtained for each individual divided by the number of affected individuals. A correlation between ADMIXTURE global ancestry fractions and RFMix summed up local-ancestry specific tracts was made for each inference with different proxy Native American references (Supplementary Figs 1–4).

#### Ethics approval and consent to participate

The data sampling protocol and the consent procedure were reviewed and approved by the State University of Paraiba Ethics committee (Protocol CAEE 51704715.4.0000.5187) and it was in accordance with the principles of Resolution 466/12 of the Brazilian National Health Council. All participants or their guardians received verbal and written explanations regarding the study procedures, and when they agreed, they signed the informed consent form and institutional declaration of approval.

## Electronic supplementary material

Supplemental Materials

## Data Availability

The datasets generated during and/or analysed during the current study are not publicly available due to ethical reasons but are available from the corresponding authors on reasonable request.
