# Supplementary material for: Origin and age of the causative mutations in KLC2, IMPA1, MED25 and WNT7A unravelled through Brazilian admixed populations
Source: Sci Rep. 2018 Nov 8;8:16552. doi: 10.1038/s41598-018-35022-1 (PMC6224410; doi:10.1038/s41598-018-35022-1)
Supplement: Supplementary file 1 — Supplemental Materials [file 41598_2018_35022_MOESM1_ESM.docx]

**Supplemental Materials**

**Title**

**Origin and age of the causative mutations in *KLC2*, *IMPA1*, *MED25* and *WNT7A* unravelled through Brazilian admixed populations.**

**Authors**

Allysson Allan de Farias^1^, Kelly Nunes^1^, Renan Barbosa Lemes^1^, Ronald Moura^2^, Gustavo Ribeiro Fernandes^3^, Uirá Souto Melo^1,4^, Mayana Zatz^1,4^, Fernando Kok^4,5+^, Silvana Santos^4,6*+^

**Affiliations**

^1^Department of Genetics and Evolutionary Biology, Biosciences Institute, University of São Paulo (USP), São Paulo, SP, Brazil.

^2^Department of Genetics, Federal University of Pernambuco (UFPE), Recife, PE, Brazil.

^3^Department of Chemistry, Institute of Chemistry, University of São Paulo (USP), São Paulo, Brazil.

^4^Human Genome and Stem-Cell Center, Institute of Biosciences, University of São Paulo (USP), São Paulo, Brazil.

^5^Department of Neurology, Faculty of Medicine (FMUSP), University of São Paulo (USP), São Paulo, Brazil.

^6^Department of Biology, State University of Paraíba (UEPB), Campina Grande, PB, Brazil.

**Supplemental Data 1 - Historical background**

The current backlands of the Northeast Brazilian territory were colonized by paleo-Amerindians approximately 375 generations ago, with an isolation of nine thousand years from European, African and Asian populations[^53^](https://paperpile.com/c/kj8xsR/vLWB). Despite the extinction of these native populations in the backlands, they were gradually incorporated, with alliances and conflicts, into European groups of colonizers throughout the eighteenth century in the states of the Northeast region[^54^](https://paperpile.com/c/kj8xsR/yaOA).

The registered documents written by chronists and voyagers, initially by French and Portuguese on the coast in the XVI century[^55^](https://paperpile.com/c/kj8xsR/Q0hE), support this trihybrid hypothesis for the Brazilian Northeast region. However, the backlands were later occupied due to a profound incursion for negotiation of cattle, promoted by Portuguese and Dutch with Sephardic Jews and Moors only in the XVII century[^56^](https://paperpile.com/c/kj8xsR/5bT6). The territories were divided as delimited land given by the King of Portugal’s authority called *sesmarias*, and the first village in the backlands was *Portalegre* in 1755. This city is near the *Martins* municipality, which revealed many affected by SPOAN syndrome, and was registered only in 1841. Most of the cities located in the Northeast backlands were originated from the Portalegre municipality led by European descendants.

The economy was developed via livestock domestication, with groups of male individuals called *sertanejos*, whereas on the coast, the main economic activity was sugarcane cultivation, with mainly African slaves and European sugar mill owners until the XIX century. The nineteenth and twentieth centuries were remarkable due to a massive influx of descendants of European and African populations in the backlands, the latter searching for economic rise and a better quality of life in the interior of the Northeast region after the prohibition of slaves negotiated by the *Lei Feijo* (1831), whereas the Native Americans in the Northeast Brazilian region were extinct, and those left over from massacres were incorporated into society through alliances.

**Supplemental Data 2 - Public Datasets**

**Simons Genome Diversity Project samples (SGDP)**

The SGDP includes 22 unrelated individuals from Africa, namely, Bantu Herero (n = 2), Bantu Kenya (n = 2), Biaka (n = 2), Esan (n = 2), Gambian (n = 2), Mandenka (n = 3), Mbuti (n = 4), Mende (n = 2), and Yoruba (n = 3). The same number of unrelated individuals from Europe are represented by Adygei (n = 1), Albanian (n = 1), Basque (n = 2), Bergamo (n = 2), Bulgarian (n = 2), English (n = 2), French (n = 3), Hungarian (n = 2), Polish (n = 1), Sardinian (n = 3), Spanish (n = 2), and Tuscan (n = 1). Additionaly, there are 22 unrelated Native Americans comprising Chane (n = 1), Karitiana (n = 3), Mayan (n = 2), Mixe (n = 3), Mixtec (n = 2), Piapoco (n = 2), Pima (n = 2), Quechua (n = 3), Surui (n = 2), and Zapotec (n = 2).

**1000 Genomes Project samples (1KGP)**

The 1KGP samples were 300 individuals distributed as 100 Yoruba from Ibadan (YRI) representing Africans, 100 Iberians (IBS) representing Europeans and 100 Han Chinese representing a population closely related Native Americans. The same number of IBS and YRI individuals were selected to run the ancestry analysis with 100 Japanese in Tokyo (JPT).


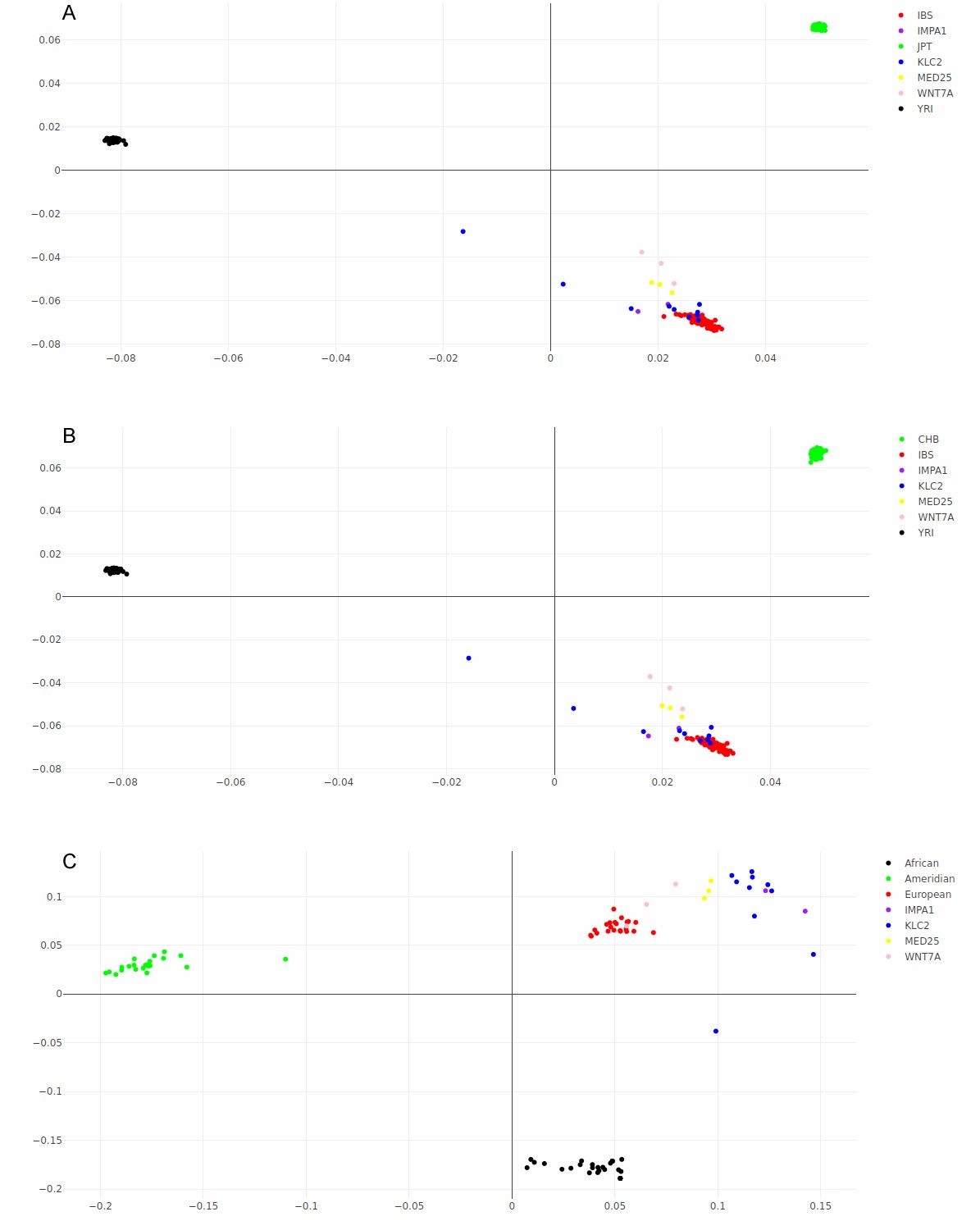


**Supplementary Figure 1** - PCA results for (a) JPT - 1KGP, (b) CHB - 1KGP, and (c) AMI - SGDP.

**
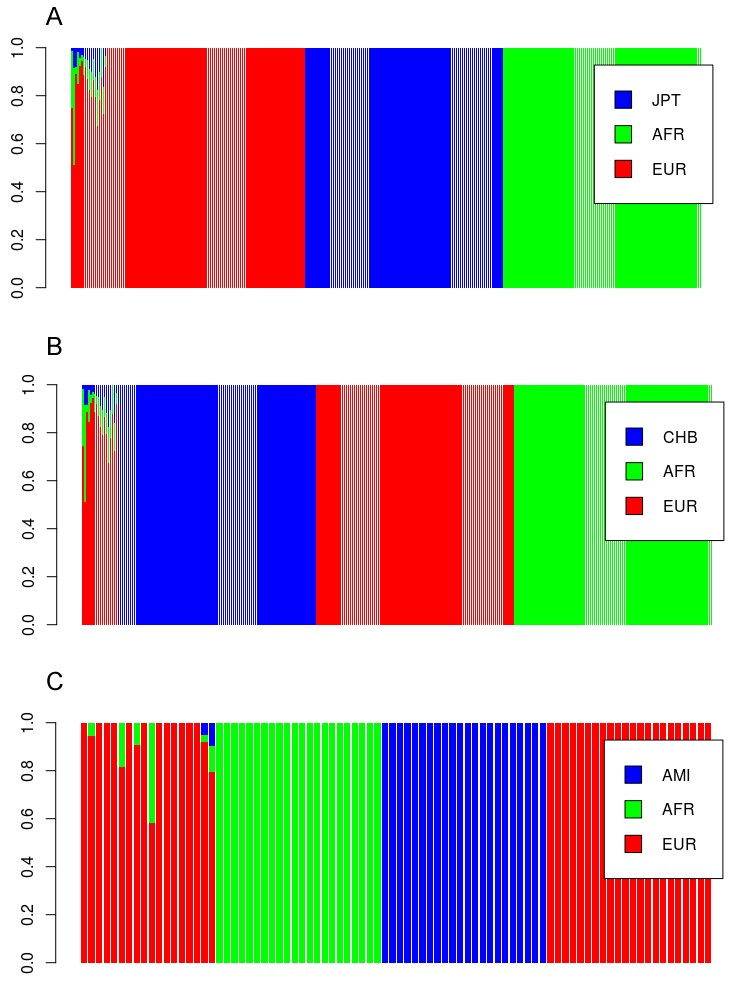
Supplementary Figure 2** - Supervised ADMIXTURE outcomes for (a) JPT - 1KGP, (b) CHB - 1KGP, and (c) AMI - SGDP.

**
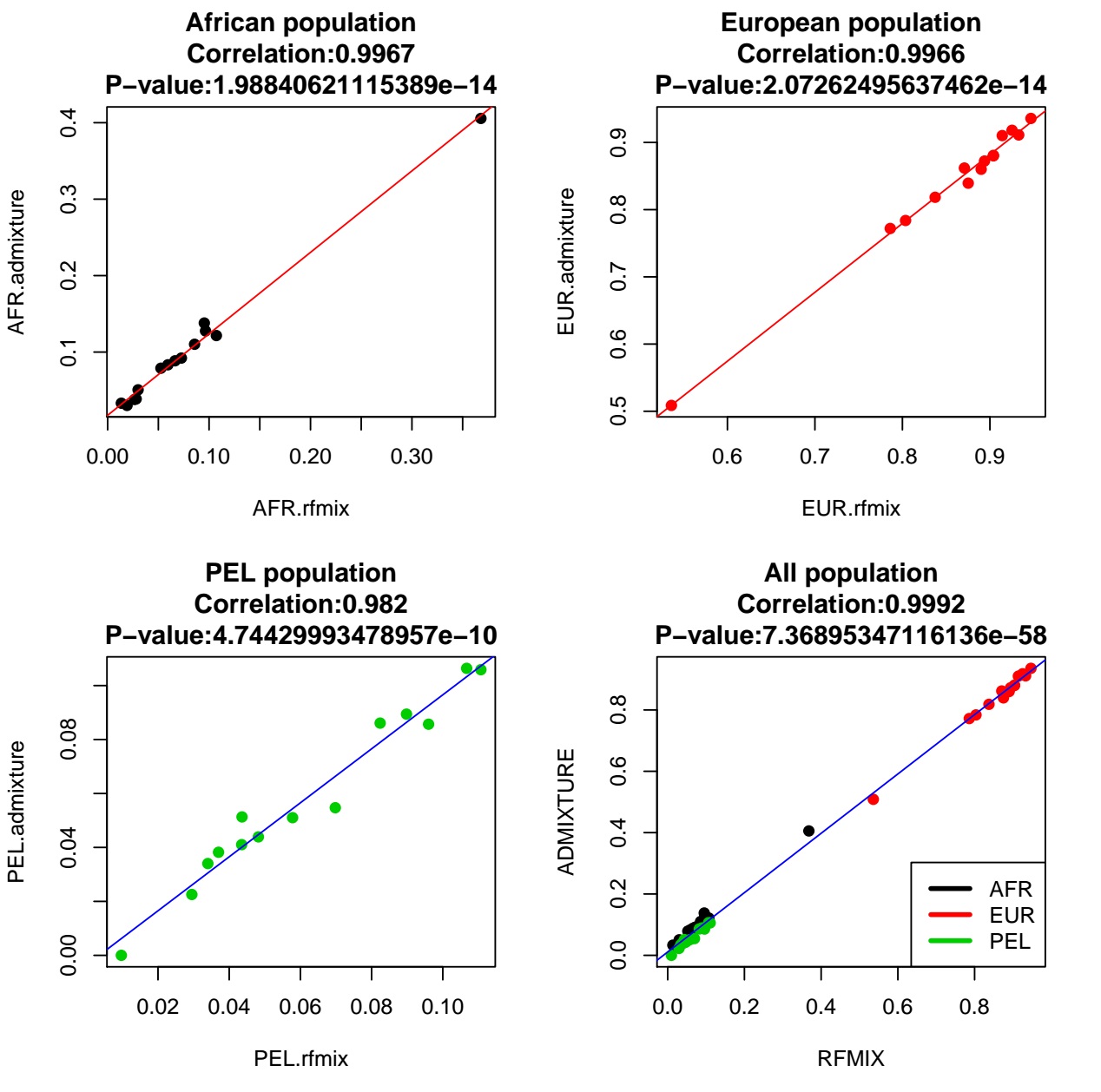
Supplementary Figure 3** - Correlation between ADMIXTURE and RFMIX results using Peruvians from Lima (PEL) as a Native American proxy.

**
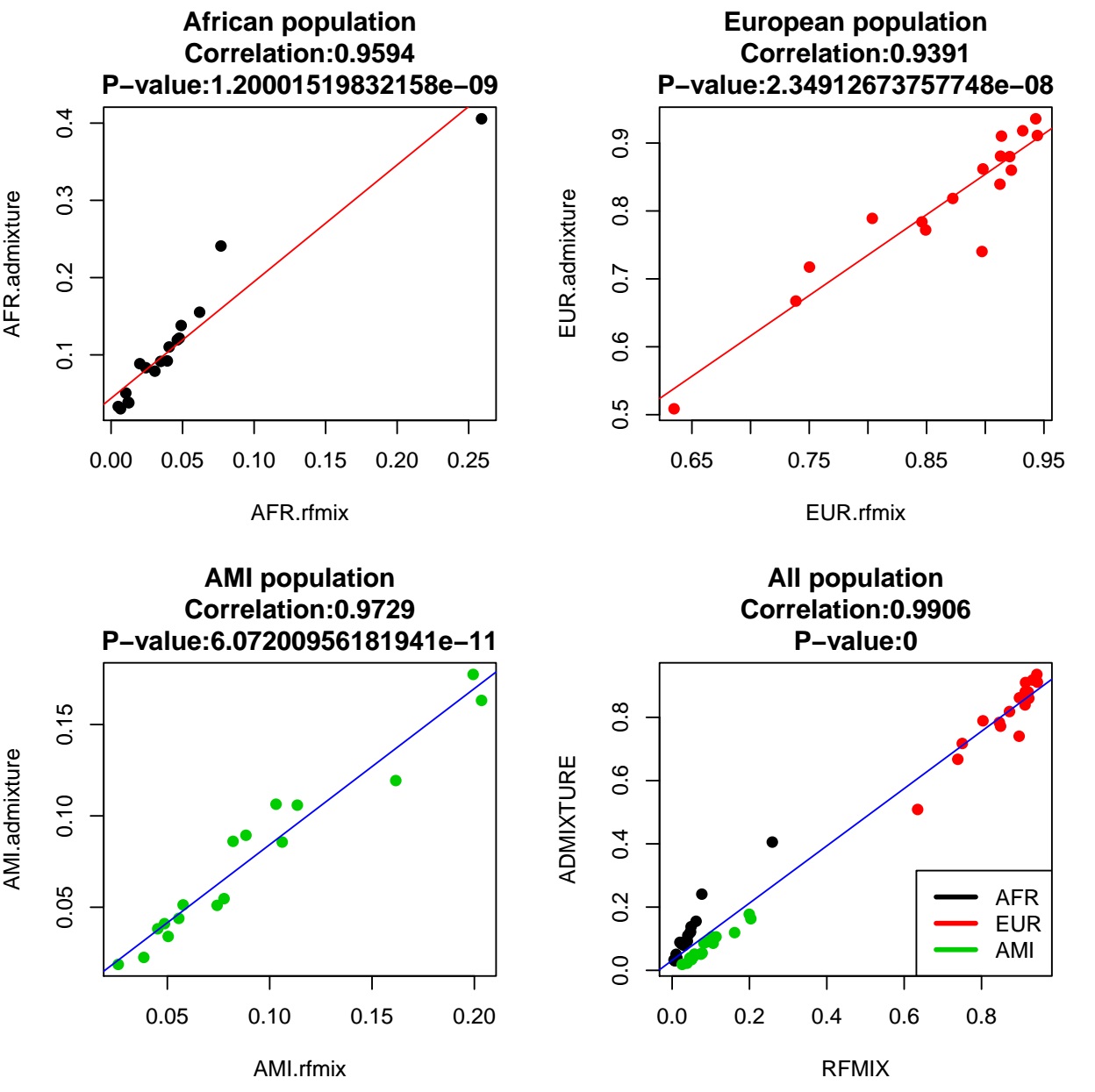
Supplementary Figure 4** - Correlation between ADMIXTURE and RFMIX results using Native American individuals data from the Simons Genome Diversity Project.

**
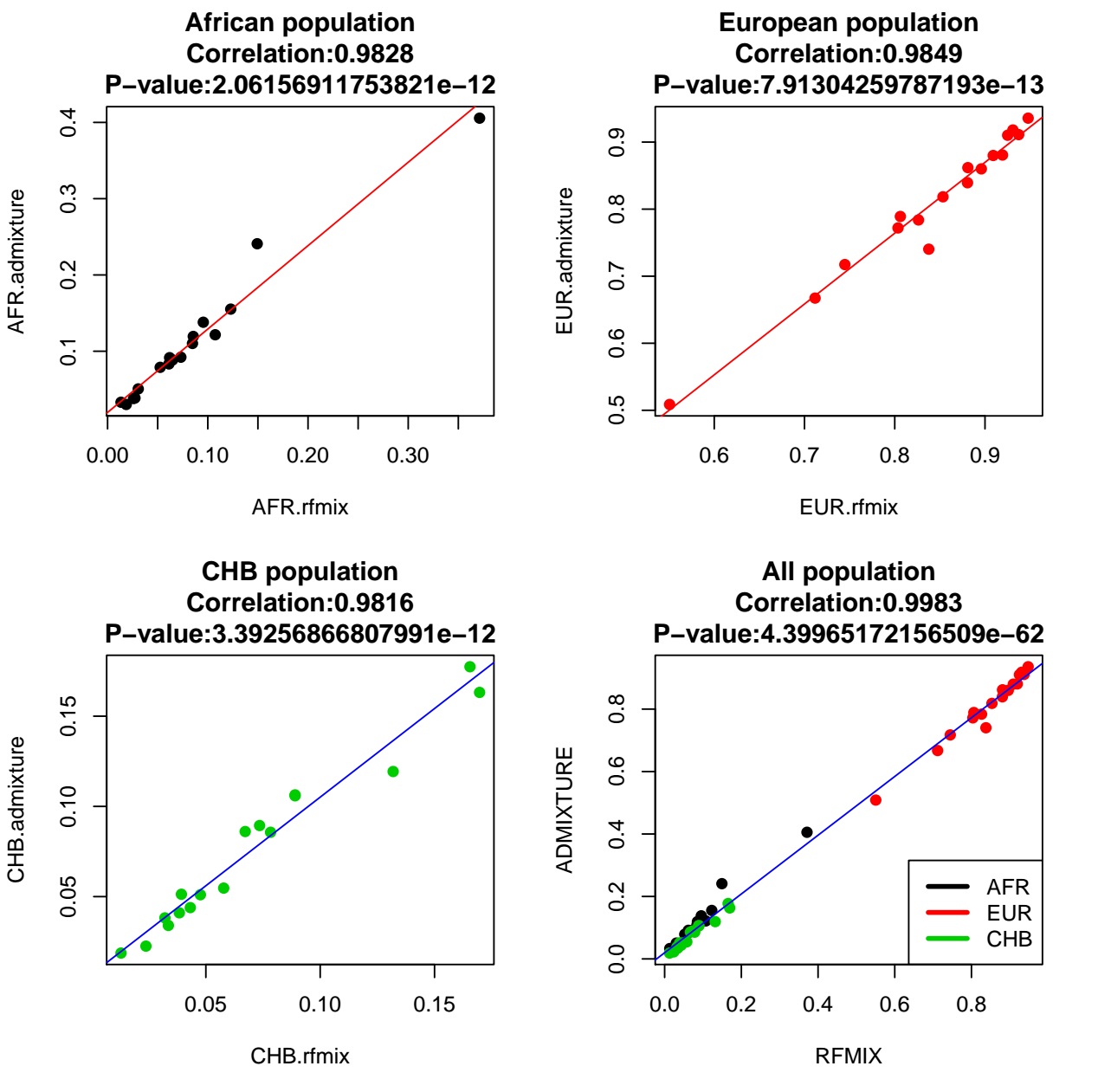
Supplementary Figure 5** - Correlation between ADMIXTURE and RFMIX result using Han Chinese (CHB) as Native American proxy.

**
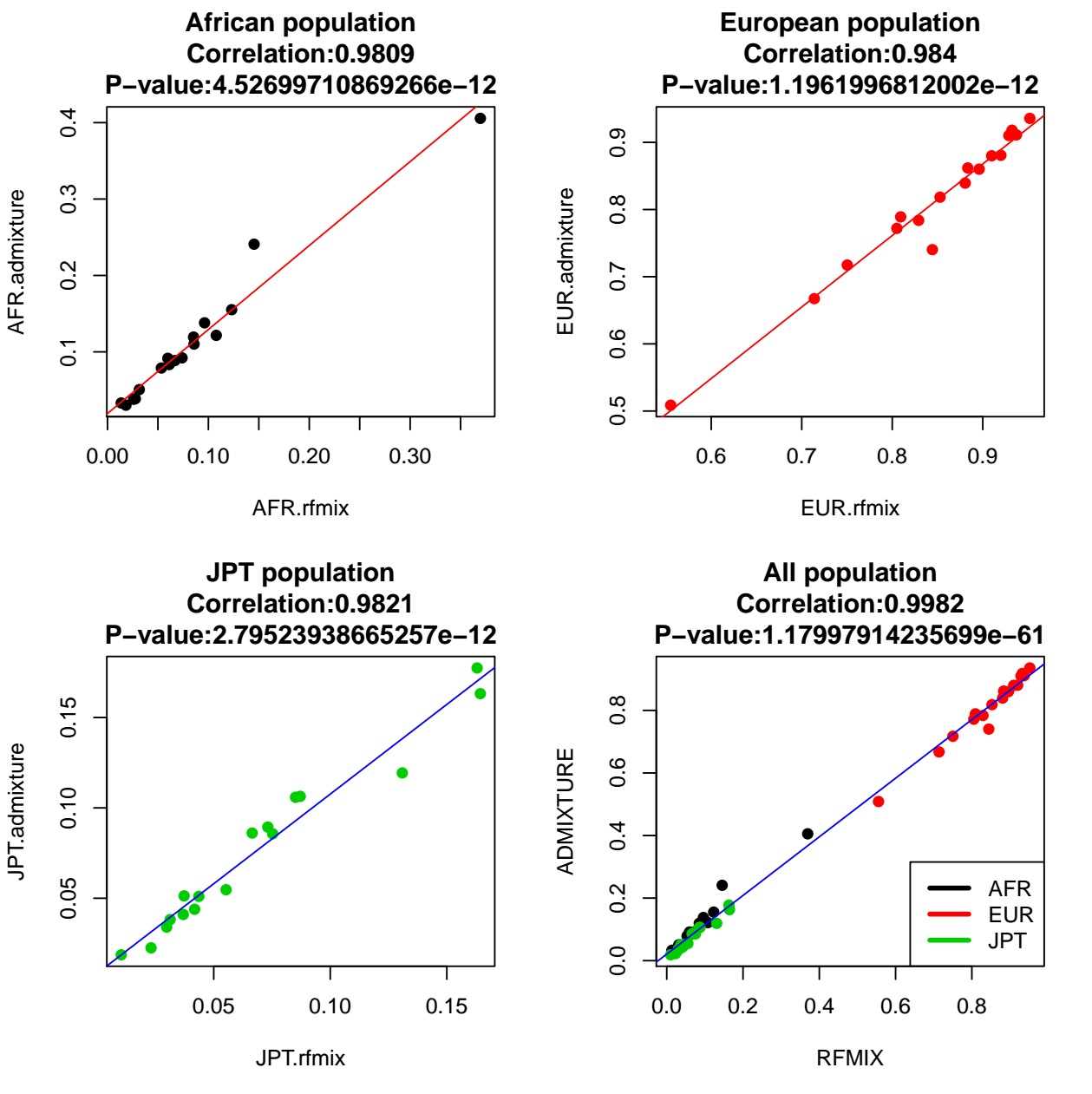
Supplementary Figure 6** - Correlation between ADMIXTURE and RFMIX results using Japanese (JPT) as a Native American proxy.

**Supplementary Table 1 -** The mean segment lengths and respective standard deviations of European and Amerindian ancestral haplotypes using three different public datasets.

|  |  | European | | | | Amerindian |
| --- | --- | --- | --- | --- | --- | --- |
|  |  | *KLC2* RN Brazilian cluster | *KLC2* World | *IMPA1* | *MED25* | *WNT7A* |
| JPT (1KGP) | Mb | 89.76(34.00) | 67.09(37.21) | 113.75(26.18) | 13.39(5.75) | 11.32(0.08) |
|  | cM | 84.89(32.76) | 60.93(43.06) | 116.12(33.67) | 34.5(7.43) | 14.27(0.09) |
|  | Mb | 78.43(16.03) | |  |  |  |
|  | cM | 72.91(16.94) | |  |  |  |
| CHB (1KGP) | Mb | 104.82(30.99) | 63.14(37.52) | 113.75(26.18) | 11.35(5.95) | 11.41(0,08) |
|  | cM | 105.23(35.76) | 60.18(45.80) | 116.12(33.67) | 31.60(14.79) | 14.47(0.09) |
|  | Mb | 83.98(29.47) | |  |  |  |
|  | cM | 82.71(31.85) | |  |  |  |
| Native American (SGDP) | Mb | 94.79(34.64) | 89.87(20.82) | 71.19(50.56) | 56.98(6.28) | 3.00(0) |
|  | cM | 96.82(39.69) | 86.51(26.8) | 75.58(59.25) | 99.33(14.58) | 4.17(0) |
|  | Mb | 92.33(3.4) | |  |  |  |
|  | cM | 91.67(7.29) | |  |  |  |
